# Pasteurization Modifies the Sensorial Attributes and Nutritional Profile of Orange Pulp By-Product

**DOI:** 10.3390/foods11131973

**Published:** 2022-07-02

**Authors:** Marta Giavoni, María José Villanueva-Suárez, Rocío De la Peña-Armada, Alejandra Garcia-Alonso, Inmaculada Mateos-Aparicio

**Affiliations:** Departamento Nutrición y Ciencia de los Alimentos, Facultad de Farmacia, Universidad Complutense de Madrid, Plaza Ramón y Cajal s/n, 28040 Madrid, Spain; marta.giavoni@gmail.com (M.G.); mjvilla@ucm.es (M.J.V.-S.); rociojim@ucm.es (R.D.l.P.-A.); alejandra.garcia.a@ucm.es (A.G.-A.)

**Keywords:** orange pulp, by-product, waste, pasteurization, nutritional profile, sensorial profile

## Abstract

After orange processing, different by-products are generated, i.e., peels, seeds and pulps. The pulp is highly perishable, being an unstable food matrix that needs a preservation process to be stored and used again in the food production chain. Pasteurization is the technique of choice before aseptically packaging and storing under refrigerated conditions. In this study, the effect of pasteurization has been evaluated on the chemical, functional and sensorial profiles. Ash content decreased (*p* < 0.05) after the thermal treatment. Indeed, magnesium, calcium and zinc diminished, although copper was found to be higher (*p* < 0.05) in the pasteurized product. Total dietary fiber decreased (*p* < 0.05), but soluble dietary fiber raised (*p* < 0.05) due to hydrolysis caused by pasteurization. SDF:IDF ratio, hydration properties, and fat binding capacity were improved. Total soluble phenolic compounds remained similar but FRAP and DPPH scavenging activity decreased (*p* < 0.05) in the pasteurized by-product. Regarding the sensorial profile, pasteurization produced darkening, appearance of a cooked smell and an increase in bitterness. Therefore, pasteurization deteriorates the sensorial profile being able to change the attributes of an added-pasteurized-pulp juice; however, it is a good choice to preserve the orange pulp by-product to formulate food products different from juices or other beverages.

## 1. Introduction

Agrofood by-products are numerous and provide a low-cost source of undervalued vegetable raw material, which is only used as livestock feed, for composting or to produce electrical or thermal energy. These by-products may also be employed as a suitable source of functional food ingredients, as they provide a large number of valuable compounds, such as dietary fiber, proteins, antioxidants, etc. [1]. The consequences of the COVID-19 pandemic crisis also affect, food systems, being important to increase their resilience. Efforts should begin by reducing postharvest and processing losses. Thus, the food industry should reduce its wastes, and at the same time, introduce innovations to offer new, acceptable, and economically viable products that can promote health and ensure food safety [2]. Indeed, there are several proposals of revalorization of by-products of plant origin and/or the recovery of their bioactive compounds [3].

Orange juice is obtained from mature oranges of, mainly, the specie *Citrus sinensis* L. (Rutaceae). The country with the largest production of oranges is Brazil with a market share of approximately 31% of world production, followed by China with a quota of about 15.8%. Concerning Europe (13.7% production) more than 6.5 million tons of oranges are harvested, being the 52.4% produced in Spain [4,5].

After orange processing, the juice and beverage companies obtain three main by-products that account for more than 50% of the entire fruit: (1) the whole peel (60–65%) that consist of the flavedo (outer peel) and albedo (spongy inner peel), (2) the pulp by-product (30–35%) that is the remnant after juice production, including cores, segment walls or membranes, juice vesicles, and seeds; and (3) seeds (<10%), sometimes removed from the flesh to produce seed oils, seeded foods and pressed cake from dried seed [6]. Peels and pulp residues are mostly a source of functional fibers and flavonoids, but are often combined into animal feed, eliminating the need for costly waste management plans. Furthermore, several recent studies have evaluated their valorization for biomethane or bioethanol production [7], however, more research to overcome toxic effects of essential oils on the microbial community is required. On the other hand, these by-products could be used in the context of producing fiber-rich and/or antioxidant food products and in the pharmaceutic and cosmetic companies (extraction of flavonoids, flavoring agents, and citric acid) [8,9,10,11], following the principles of the circular economy and the Sustainable Development Goals (SDGs), mainly 12 (responsible consumption and production). The problem to follow the mentioned premises is the large quantity available within the juice industry, and their perishable character. They are instable food matrixes that needs a process of preservation–stabilization to be re-introduced into the food production chain. Focusing on the orange pulp, part of this by-product undergoes pasteurization, and it is aseptically packed and stored under chilled conditions. This way of preservation allows part of orange pulp to be used in food production, mainly to increase the solids content to give body to pulpy juices and other similar beverages. However, pasteurization is a thermal treatment that may affect the bioactive and organoleptic compounds present in orange pulp, and consequently affect the final product [3,12].

The purpose of the present study was to explore the impact of the pasteurization on the orange pulp by-product. The parameters evaluated included a nutritional and functional characterization as well as a sensorial analysis to find out to what extent pasteurization may affect the perception at the sensory level, which refers to sight, smell, and taste.

## 2. Materials and Methods

### 2.1. Samples

The orange trees are grown in Valencia (Spain) in areas with permeable, slightly calcareous soils. The oranges (*Citrus sinensis* L.), Navel variety, are handpicked in late summer and early autumn. These fruits are processed for preparation of orange juice by squeezing the oranges. Subsequently, the juice is separated by sieving, generating a by-product consisting of the edible part of the named, sound, ripe and fresh fruit, namely orange pulp by-product, which was provided in January–February 2019 by ZUVAMESA (Valencia, Spain). Samples were received aseptically packed and in chilled conditions (≤4 °C) as non-pasteurized and pasteurized pulp (NPP and PP). The accomplished pasteurization at ZUVAMESA achieved the inactivation of enzymes and the destruction of microorganisms. Part of the samples were frozen (−20 °C), and defrosted for analysis of moisture, soluble solids, pH, acidity, and ash in fresh sample, and other part was freeze-dried (Freeze-dryer Telstar, mod. LyoQuest, Terrassa. Spain) when it was received in the laboratory. Afterwards, they were homogenized by grinding until a fine powder is obtained (<1 mm) and stored in vacuum recipients at room temperature prior to analysis (*n* = 4) during 2019–2020.

### 2.2. Chemical Characterization

Moisture was determined by lyophilizing at Telstar, mod. LyoQuest. The total soluble solids were quantified with a digital refractometer (Atago RX-1000, Tokyo, Japan) at 20 °C. pH was determined with a pH-meter (Microph 2000, Crison, Barcelona, Spain) in the supernatants after centrifugation of the samples at 3024× *g*, 10 min. Acidity was analyzed by titration with NaOH 0.1 N. Formaldehyde was added to the resulting liquids from acidity determination and the formol index was measured by titration with NaOH 0.1 N. Ash content was determined by incineration in a muffle furnace (Milestone mod. MLS-1200 Pyro, Bangbi, Denmark) with the following conditions: 250 °C, 30 min; 500 °C, 15 min; 550 °C, 15 h. Macro- and microelements were measured in the resulting ashes with a PerkinElmer Analyst200 atomic absorption spectrophotometer (Waltham, MA, USA) according to the procedure reported by Mateos-Aparicio et al. [13].

### 2.3. Functional Characterization

Dietary fiber was determined with AOAC method 991.43 [14] without the step of the heat stable alpha-amylase, which was not used because of this by-product has not starch and to avoid the losses due to the high temperatures of the treatment with this enzyme (100 °C, 30 min). Thus, it consisted of a protease treatment (pH 7.5, 60 °C, 30 min) followed by an amyloglucosidase treatment (pH 4.5, 60 °C, 30 min). After enzymatic treatment, some samples were filtered to obtain insoluble dietary fiber (IDF), and others were added ethanol to precipite soluble dietary fiber and filtered to determine total dietary fiber (TDF). In both residues, TDF and IDF, protein and ashes contents were analyzed to calculate the dietary fiber content. 

The swelling capacity, water retention capacity and oil retention capacity were determined in both samples. The swelling capacity, and water and oil retention capacity were carried out according to Robertson et al. [15]. Briefly, for swelling capacity, a known weight of the dry sample was hydrated in a graduated cylinder, covered, and left undisturbed at room temperature for 18 h. Subsequently, the settled volume occupied by the sample was recorded. Regarding water retention capacity, a known weight of the dry sample was hydrated in a centrifuge tube for 18 h. After that, supernatant was separated by centrifugation (3024× *g*; 20 min) from the insoluble residue and discarded. Finally, the weight of the hydrated residue and the dry residue was recorded. The oil retention capacity was determined as WRC, but the water was replaced with extra virgin olive oil.

The determination of vitamin C [16] and organics acids [17] was carried out using a Phenomenex Luna RP-C18 column (250 × 4.6 mm, 5 μm, Phenomenex, Torrance, CA, USA) in Agilent 1100 Series chromatograph (Agilent Technology, Palo Alto, CA, USA). The samples were blended with 4.5% metaphosphoric acid and the mixture was homogenized for 5 min with Omni Mixer Homogenizer model 17,106 (Kennesaw, GA, USA) and centrifuged at 3024× *g*, 20 minusing a Hettich zentrifugen Universal 320 (Buckinghamshire, England). The supernatants were filtered through a 0.45-μm Millex membrane (Millipore, Molsheim, France) in order to separate the dispersed solid particles. The mobile phase was Milli-Q water acidified with H_2_SO_4_ 12 M on isocratic gradient and flow rate 0.9 mL/min. The oven temperature was 25 °C and DAD set at 245 nm for vitamin C and at 215 nm for organic acids.

Polyphenols were recovered from the freeze-dried samples by extracting them with methanol:water (50:50 *v*/*v*) acidified to pH 2 with 2 M HCl and acetone:water (70:30 *v*/*v*). The Folin–Ciocalteu procedure was used to quantify the total extractable polyphenols content (TPC). TPC as well as ferric reducing antioxidant power (FRAP) and DPPH radical-scavenging activity was monitored by spectrophotometry (UV/Vis Lambda EZ210; Perkin–Elmer, Jügesheim, Germany). FRAP was used to evaluate the reducing power of the polyphenol extracts. Increase in absorbance due to the formation of a colored 2,4,6-tri(2-pyridyl)-1,3,5-triazine-Fe^2+(^TPTZ-Fe^2+^) complex was monitored at 595 nm. A Trolox and an ascorbic acid reference curves (0.1–1 mmol·L^−1^) were used (r^2^ = 0.997; r^2^ = 0.999). The DPPH radical-scavenging activity of the polyphenolic extracts was assayed with 250 µL of sample solution at different concentrations (20–0.0125 mg mL^−1^) mixed with 1000 µL of methanolic DPPH solution (100 µmol L^−1^). Different concentrations of ascorbic acid (20–0.0125 mg mL^−1^) were used as a positive control for comparison. The mixture was shaken and kept at room temperature in the dark. Absorbance was measured at 517 nm after 30 and 60 min in the samples and the positive control [18].

### 2.4. Sensorial Characterization

#### 2.4.1. Color Analysis

Color analysis was carried out with a Minolta CR-200 Chroma Meter (Minolta, Osaka, Japan) as L*, a*, b* values. NPP and PP by-products were set in a small cylindrical glass with a lid. Measurements were done on the top and the base of the glass and directly on the pulp by-products. 

#### 2.4.2. Sensory Profile

Three different sensorial analyses, i.e., descriptive, discriminative and affective tests, were carried out with an untrained panel composed of 87 non-trained members, 48 females and 39 males non-smokers aged 18–39. The majority (71%) of them were regular consumers of orange juice (65% natural and 35% commercial juices). Samples were served in similar amount in transparent glasses listed with a three-digital number. Glasses were placed on white paper to achieve a uniform background. Tap water and unsalted breadsticks were given to the panelists to clean their palate between tastings. Data were reported as the mean of all the scores. 

For the descriptive test, the panel assessed the following attributes: color, aroma, flavor, aftertaste and texture using a 9-point scale. Regarding color, panelist had to classify the samples between yellow, orange or reddish, once classified they scored the tone and intensity. The discriminative pair comparison test consists of the comparison between both by-products pointing which one was greater intensity regarding color, fresh and cooked smell, sweetness, acidity and bitterness. Regarding the affective test, panelists had to choose which by-product they preferred and reply about the preference.

### 2.5. Statistical Analysis

Data are shown as the means ± standard deviation (*n* = 4). One-way ANOVA was used for statistical analysis of the data. Differences were significant at *p* < 0.05 (Statgraphics Centurion XVI, Warrenton, VA, USA).

## 3. Results and Discussion

### 3.1. Chemical and Functional Evaluation

The amount of water, ashes and pH were higher (*p* < 0.05) in non-pasteurized pulp (NPP), meanwhile °Brix were higher (*p* < 0.05) in the pasteurized pulp (PP) (Table 1). The pasteurization process involves a partial evaporation of water, which may explain the difference in the higher soluble solids value of PP. The organic acids, i.e., citric and malic, and the vitamin C (ascorbic acid) were identified and found in similar amount independently of the pasteurization (Table 1). Vitamin C is thermolabile, however, it was not observed a significant loss in PP. Similar results were found in different juices and other beverages [19,20,21]. Results differ from the physicochemical stability study of *Citrus sinensis* (L.) Osbeck cv. juice during frozen storage analyzed by Giuffrè et al. [22]. The study concluded that five months of frozen storage decreased the pH, °Brix, formol number and vitamin C content, and acidity values remained stable. Hence, temperature seems to be a crucial factor in *Citrus sinensis* (L.) processing. 

The analysis of macro- and microelements in the ashes of the samples (Table 2) showed changes in calcium, magnesium and zinc that appeared in higher amount (*p* < 0.05) in NPP than in PP; conversely, copper appeared in lower amounts (*p* < 0.05) in the NPP. It has been previously observed that the mineral content changes when thermal treatments are applied, releasing protein-bound minerals and/or changing the levels of some inhibitors including oxalates, phytates, tannins and phenolic compounds [23,24]. However, Sádecká et al. [25] and Andrés et al. [16,17] did not find any change in orange juice and milk- and soy-smoothies, respectively; neither Benítez et al. [26] in onion by-products.

Total dietary fiber (TDF) content was significantly higher (*p* < 0.05) in NPP (Table 1) than in PP because of the insoluble fraction (IDF) amount; however, the soluble fraction (SDF) was higher (*p* < 0.05) in PP than in NPP. *Citrus sinensis* L. by-products are sources of dietary fiber, but are commonly used in animal feed. However, pulp by-product presents a remarkable fiber content that can be employed as a functional ingredient to formulate food products. Indeed, they represent the most important raw material for producing commercial pectin (85.5%), with apple by-product next (14.0%), sugar beet last (0.5%). The pasteurization process carried out to stabilize the products destroys part of the matrix affecting dietary fiber. In fact, TDF content of several vegetables, such as carrots, potatoes, peas, etc., was reduced by applying different thermal methods [27,28,29]. On the other hand, the pasteurization process can solubilize dietary fiber, improving soluble/insoluble fiber ratio (Table 1) as it was observed before in onion by-products [18]. Thus, PP is a value-added-product due to the major SDF compared to NPP. In fact, SDF can be fermented in the colon positively affecting microbiota, generating a major prebiotic potential [30]. The increase in SDF could be caused by the partial hydrolysis and breakdown of cellulose, hemicellulose and/or pectins induced by the heat treatments which can lead to the solubilization of low and medium molecular weight polysaccharides and oligosaccharides as well as the loss of lignin.

**Figure 1:**
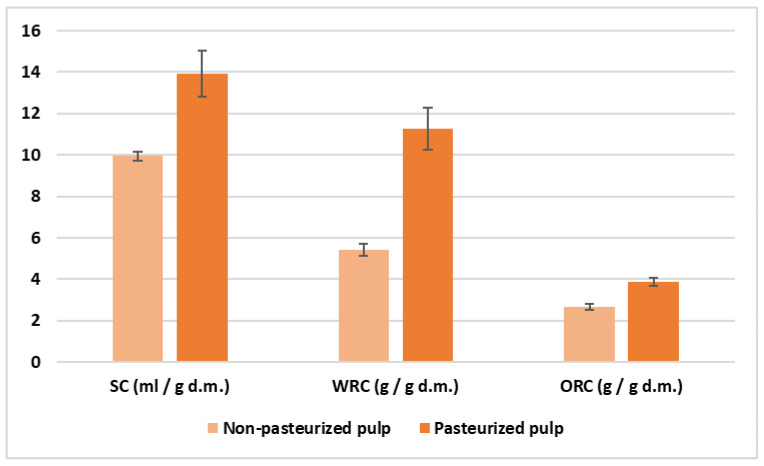
Physicochemical properties of non-pasteurized and pasteurized orange pulp by-products. SC: Swelling capacity; WRC: Water retention capacity; ORC: Oil retention capacity; d.m.: dry matter.

The physicochemical properties analyzed, that is, swelling, water retention and oil retention capacities (SC, WRC and ORC, respectively), were higher (*p* < 0.05) in PP than in NPP (Figure 1), showing better hydration properties and capacity to bind oil of PP. This last property makes this pasteurized by-product suitable to stabilize fat-rich food and emulsions. Thus, pasteurization can improve the technological aptitude of orange pulp.

**Figure 2:**
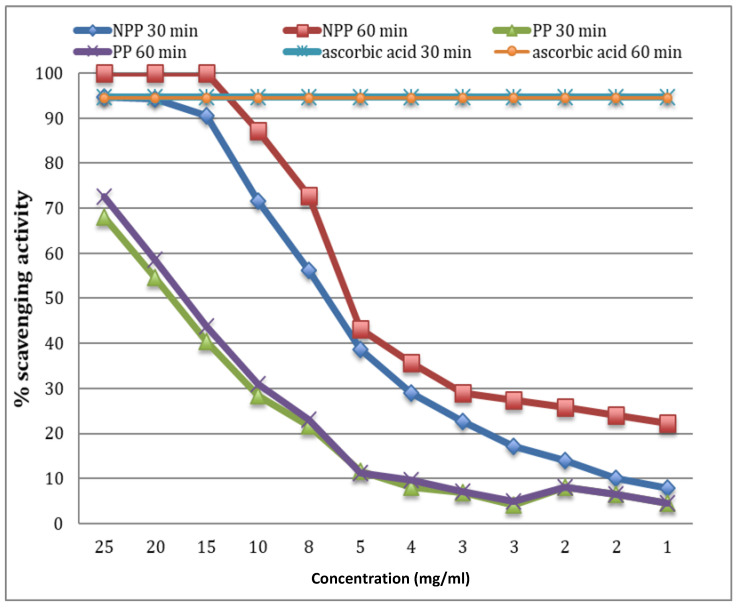
Scavenging activity on DPPH radical of ascorbic acid and extractable polyphenols from non-pasteurized pulp (NPP) and pasteurized pulp (PP) at 30 and 60 min.

TPC of the samples were similar (NPP: 5.72 ± 0.21; PP: 5.44 ± 0.13 mg gallic acid/g d.m). However, Agcam et al. [31] observed an enhancement of the total phenolic concentration after heat pasteurization treatments. Furthermore, two different assays were performed in order to address the antioxidant capacity of both samples, namely, FRAP and DPPH. These two methods had been suggested as appropriate by numerous authors for measuring the antioxidant activity of fruit juice extracts, and therefore should be accurate for orange pulp [32]. FRAP was higher (*p* < 0.05) in NPP (62.33 ± 3.22 µg Trolox/g d.m.) than in PP (46.58 ± 2.16 µg Trolox/g d.m.). Furthermore, the scavenging activity on DPPH radical was tested in a range of concentration from 1 mg/mL to 25 mg/mL and compared with the activity of ascorbic acid at 30 and 60 min (Figure 2). The DPPH was totally scavenged for NPP at concentrations ranged 25–20 mg/mL, and greater than 90% at 15 mg/mL. However, the scavenging activity was 68% at 25 mg/mL for PP, and it was less than half (40.4%) at 15 mg/mL. The half maximal effective concentration (EC50) was minor (*p* < 0.05) for NPP (9.2 mg/mL at 30 min and 6.7 mg/mL at 60 min) than for PP (18.5 mg/mL at 30 min and 17.4 mg/mL at 60 min). Therefore, the antioxidant power of NPP is higher than in PP indicating a loss of activity as a negative effect during the pasteurization. It is remarkable that the antioxidant activity decrease is not due to vitamin C and/or extractable polyphenols because these compounds are not significantly diminished after pasteurization. Oliveira et al. [33] observed that pasteurized peaches presented similar amounts of total phenolics, but an increase of their antioxidant activity; maybe due to a major availability of carotenoids and the formation of new products. Bhattacherjee et al. [34] reported similar amounts of vitamin C and total polyphenol contents in pasteurized aonla juice; however, they observed changes in individual antioxidant compounds. Jeong et al. [35] observed that thermal treatment of citrus peels can liberate bound phenolics, leading to an increase in the total phenolic content (TPC) after the processing. Thus, pasteurization degrades some components but improves the availability of others and can generate new compounds, different from those in the fresh samples, mainly through reactions such as the Maillard reaction.

### 3.2. Sensorial Evaluation

The lightness (L*) was non-significant being 58.6 ± 0.9 in NPP and 58.9 ± 0.3 in PP. The chroma a* was slightly significant (*p* < 0.05) between NPP (1.1 ± 0.2) and PP (1.7 ± 0.3), and chroma b* was found significantly higher (*p* < 0.05) in NPP (46.5 ± 2.3) than in PP (35.6 ± 4.0). Results differ from the orange juice study reported by Velázquez-Estrada et al. [36] in which, after the pasteurization process (90 °C/1 min), no significant differences were found between untreated and treated in terms of color parameters (L*, a*, b*).

**Figure 3:**
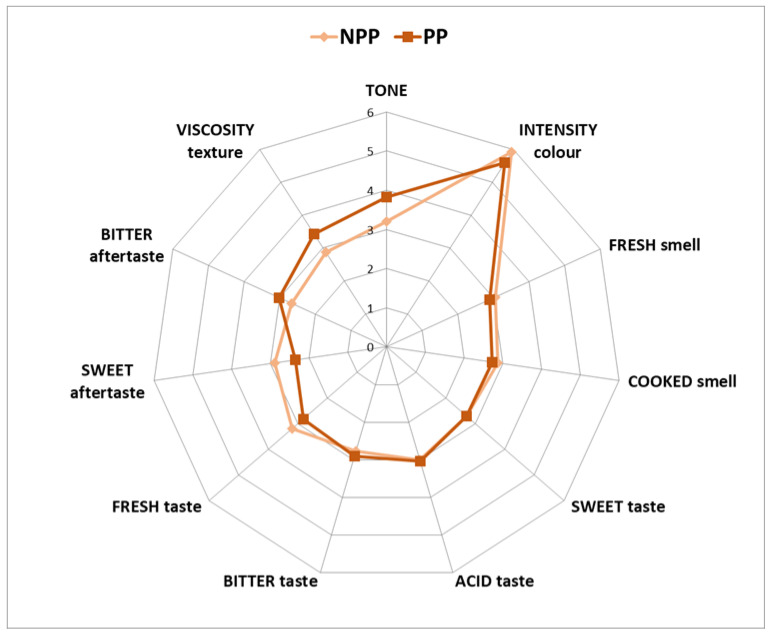
Radial chart about the sensorial attributes of non-pasteurized pulp (NPP) and pasteurized pulp (PP) evaluated by sensory panel.

In descriptive analysis (Figure 3), the tone color was higher (*p* < 0.05) scored for pasteurized sample than for non-pasteurized one; however, the color intensity was similar. NPP was classified as yellow while PP was described as orange, which suit perfectly with the analysis made with CIE L* a* b* color system where NPP shows a color more yellowish than the pasteurized one. Pasteurization could lead to the formation of high molecular weight compounds of brown color, called melanoidins, after the Maillard reaction, which can darken the pasteurized by-product [37]. Regarding the smell, the panelists related NPP with a fresher and less cooked smell than PP, although no significant differences were found. It makes sense because the Maillard reaction is responsible for the typical cooked smell in foods. In the case of taste, acidity and sweetness were scored similarly in both samples; however, PP was perceived as slightly more bitter and significantly less fresh (*p* < 0.05) than NPP. The most remarkable differences were found in aftertaste parameters, as NPP was described as sweeter (*p* < 0.05) and less bitter (*p* < 0.05) than PP. Similar to many fruits, the flavor of freshly squeezed orange juice is mainly related to aldehydes (e.g., acetaldehyde, hexanal, octanal, decanal, neral, geranial, and Z-hex-3-enal) and esters (e.g., ethyl butanoate and ethyl-2-methylpropanoate), plus a smaller number of alcohols (e.g., linalool), ketones (1-octen-3-one), and hydrocarbon terpenes (myrcene, β-pinene, and possibly limonene). By heating, the levels of reactive aroma impact compounds, such as neral and geranial, are reduced and off-flavors or their precursors are created from Maillard, Strecker, and acid catalyzed hydration reactions [38]. Off-flavors such as 4-vinylguaiacol, p-cymene, and carvone are the chemical reactions products [39]. Pasteurization decreases the content of the majority of terpene alcohols, esters, aldehydes, ketones and sesquiterpenes in pulpy orange juice, as a consequence of their thermolability, which regarded, in particular, sesquiterpenes. On the other hand, pasteurization increases some volatile monoterpenes, e.g., of α-pinene, α-thujene, α-phellandrene, δ-3-carene, α-terpinene, d-limonene, cis-β-ocimene, γ-terpinene and 4-carene [25]. The texture of PP sample was described as more viscose (*p* < 0.05) than the NPP sample. 

**Figure 4:**
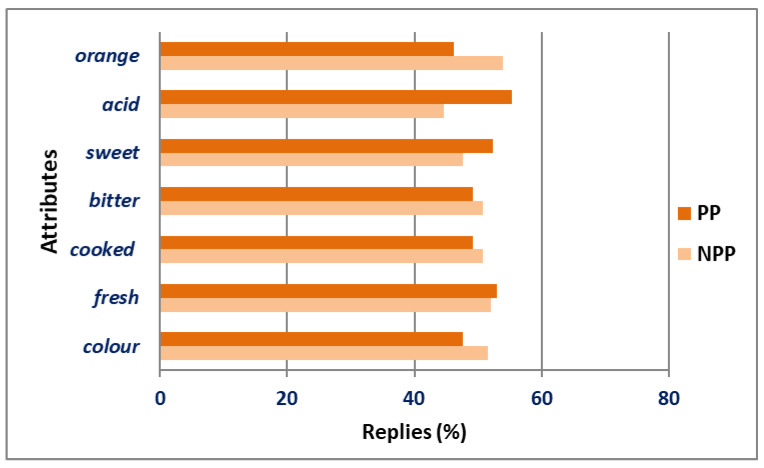
Discriminative results of attributes from non-pasteurized pulp (NPP) and pasteurized pulp (PP) evaluated by sensory panel.

The discriminative test performed consisted of presenting the two samples to the panelists, and asking them to identify which of the two samples had an outstanding attribute over the other sample, such as which of the two samples was more acidic. Figure 4 represents the percentages of replies related to the evaluated attributes (similar to orange fruit, acidity, sweetness, bitterness, similar to cooked flavor, freshness, and similar to orange color). Among the evaluated attributes, the panelist were only able to discriminate two, namely, lower acidity and similarity to orange fruit in the case of NPP. The other attributes were not well differentiated by the panelists, but this was to be expected because the panelists were not trained in this type of analysis. In fact, this untrained panel was used because we sought feedback from regular consumers to see the possibilities of using the pasteurized ingredient for food production without affecting the expected sensory characteristics. In the affective part of the sensory analysis, panelists preferred the color (59%), smell (56%) and taste (62%) of NPP.

## 4. Conclusions

After the juice extraction, the orange pulp is a remnant that usually derived to animal feed, although it is often added to pulpy juices. In this last case, pasteurization is the technique of choice to stabilize this ingredient. This process decreases antioxidant activity and total dietary. However, there are no major changes in vitamin C and organic acids, and it produces a dietary fiber solubilization that improves physico-chemical properties generating a functionalized ingredient to formulate new food products. 

Regarding sensorial analysis, the color, smell and taste are better rated in non-pasteurized sample, and it was also preferred by the panelists because it reminds of fresh orange. Therefore, the search for procedures to preserve juices and to stabilize pulps (by-products) used to add in pulpy juices should focus on decreasing the negative impact on aromatic compounds caused by thermal treatments as pasteurization. However, pasteurization is a cheap and a good option to stabilize and functionalized orange pulp to be used in other products different to beverages.

## Figures and Tables

**Table 1:** Characterization of non-pasteurized (NPP) and pasteurized (PP) orange pulp by-product.

	Non-Pasteurized Pulp	Pasteurized Pulp
Moisture (g water/100 g f.m.)	86.2 ± 0.2 *	83.7 ± 0.4
Brix (°degree)	12.6 ± 0.0	12.9 ± 0.0 *
pH	3.8 ± 0.1 *	3.6 ± 0.0
Acidity (g citric acid/100 mL)	0.5 ± 0.1	0.7 ± 0.0 *
Formol Index (ml NaOH/100 mL)	18.5 ± 0.7	18.0 ± 0.0
Minerals (g ashes/100 g d.m.)	3.2 ± 0.1 *	2.7 ± 0.1
Ascorbic acid (mg/100 g d.m.)	17.63 ± 0.62	16.46 ± 0.64
Citric acid (mg/100 g d.m.)	286.58 ± 22.99	328.96 ± 24.94
Malic acid (mg/100 g d.m.)	74.78 ± 6.91	61.08 ± 6.73
Insoluble dietary fiber (IDF) (g/kg d.m.)	19.2 ± 0.94 *	7.3 ± 0.5
Soluble dietary fiber (SDF) (g/kg d.m.)	8.9 ± 2.2	13.1 ± 0.9 *
Total dietary fiber (TDF) (g/kg d.m.)	27.1 ± 2.0 *	20.4 ± 1.0
SDF:IDF	0:5	1:8
SDF:TDF	0:3	0:6

f.m.: fresh matter; d.m., dry matter. * significantly higher (*p* < 0.05). *n* = 4.

**Table 2:** Macro- and microelements of non-pasteurized (NPP) and pasteurized orange pulp (PP) by-products.

	Non-Pasteurized Pulp	Pasteurized Pulp
Macroelements (mg/g)		
Ca	1.38 ± 0.16 *	0.54 ± 0.03
K	2.33 ± 0.09	2.44 ± 0.30
Na	0.88 ± 0.23	1.40 ± 0.60
Mg	0.42 ± 0.05 *	0.20 ± 0.02
Microelements (µg/g)		
Cu	3.91 ± 0.82	6.10 ± 0.38 *
Fe	26.96 ± 0.45	26.78 ± 0.35
Mn	2.82 ± 0.04	2.14 ± 0.07
Zn	13.73 ± 0.82 *	11.42 ± 0.38

* significantly higher (*p* < 0.05), *n* = 4.

## Data Availability

The datasets used and/or analyzed during the current study are available from the corresponding author on reasonable request.
